# Two TaqMan real-time quantitative PCR assays for the detection of Alongshan virus, a new member of the tick-borne Flaviviridae family

**DOI:** 10.1099/acmi.0.000917.v3

**Published:** 2026-02-09

**Authors:** Onya Opota, Florian Tagini, Valentin Loup, Zahera Dance, Valeria Cagno, Jakub Kubacki, Gilbert Greub

**Affiliations:** 1 Institute of Microbiology, Lausanne University Hospital and University of Lausanne, Lausanne, Switzerland; 2 Swiss National Center for Tick-Borne Infections, Lausanne, Switzerland; 3 Infectious Disease Service, Lausanne University Hospital and University of Lausanne, Lausanne, Switzerland; 4 Institute of Virology, Vetsuisse Faculty, University of Zurich, Zurich, Switzerland

**Keywords:** Alongshan virus (ALSV), diagnostic PCR, emerging pathogens, flavivirus, meningoencephalitis, real-time quantitative PCR, tick-borne infections, viral infections

## Abstract

**Introduction.** Alongshan virus (ALSV) is a tick-borne *Flaviviridae*. It has been detected in *Ixodes* ricinus and *Ixodes persulcatus* across China, Russia, Finland, Switzerland and Germany.

**Hypothesis/Gap Statement.** However, the clinical relevance and the pathogenicity of ALSV in humans remain unclear. Sensitive and specific molecular tools are needed to support surveillance and to enable clinical investigations of ALSV in suspected cases of tick-borne meningoencephalitis.

**Aim.** We aimed to develop, validate and integrate two ALSV-specific TaqMan real-time quantitative PCR (qPCR) assays on our open, high-throughput molecular diagnostic platform.

**Methodology.** We designed assays targeting conserved regions of the NS3 (helicase-protease) and NS5 (RNA-dependent RNA polymerase) genes, incorporating degenerate bases and locked nucleic acid modifications where needed to accommodate documented viral diversity and to harmonize the annealing temperature with TaqMan probe-related technologies and our platform. Analytical sensitivity and reproducibility were assessed using synthetic plasmids carrying the targets; specificity was evaluated against 41 cerebrospinal fluid (CSF) pathogens and 30 winter CSF specimens from patients with suspected central nervous system infection. ALSV-positive Swiss tick extracts served as biological positives.

**Results.** Detection frequencies for NS3 PCR were 100%, 100%, 92%, 72%, 20%, 28 and 0% at 1,000, 100, 10, 5, 2, 1 and 0.1 copies per reaction, respectively. For NS5, the detection frequencies were 100%, 100%, 92%, 88%, 40%, 20% and 0% at the same concentrations. Using *a priori* definition of limit of detection (LoD) as ≥95% positive replicates, LoD was 100 copies per reaction for both real-time qPCRs. However, as the PCRs are performed in triplicate in our platform, the LoD can be estimated at five copies per reaction for the NS3 real-time qPCR and two copies per reaction for the NS5 PCR. Intra- and inter-run reproducibility across five independent runs met diagnostic standards. Specificity was 100% (71/71). ALSV-positive tick samples were detected by both assays, with lower cycle thresholds for NS5.

**Conclusions.** We validated two ALSV real-time qPCR assays suitable for integration into open molecular diagnostic platforms. These assays enable syndromic testing alongside other encephalitis-associated viruses (e.g., Tick-borne encephalitis virus and West Nile virus) and will facilitate timely clinical management of suspected cases, high-throughput tick surveillance and future clinical studies of potential ALSV pathogenic role.

## Data summary

All data, reagents and software required for our work to be reproduced are described in the manuscript.

## Introduction

**Fig. 1.**
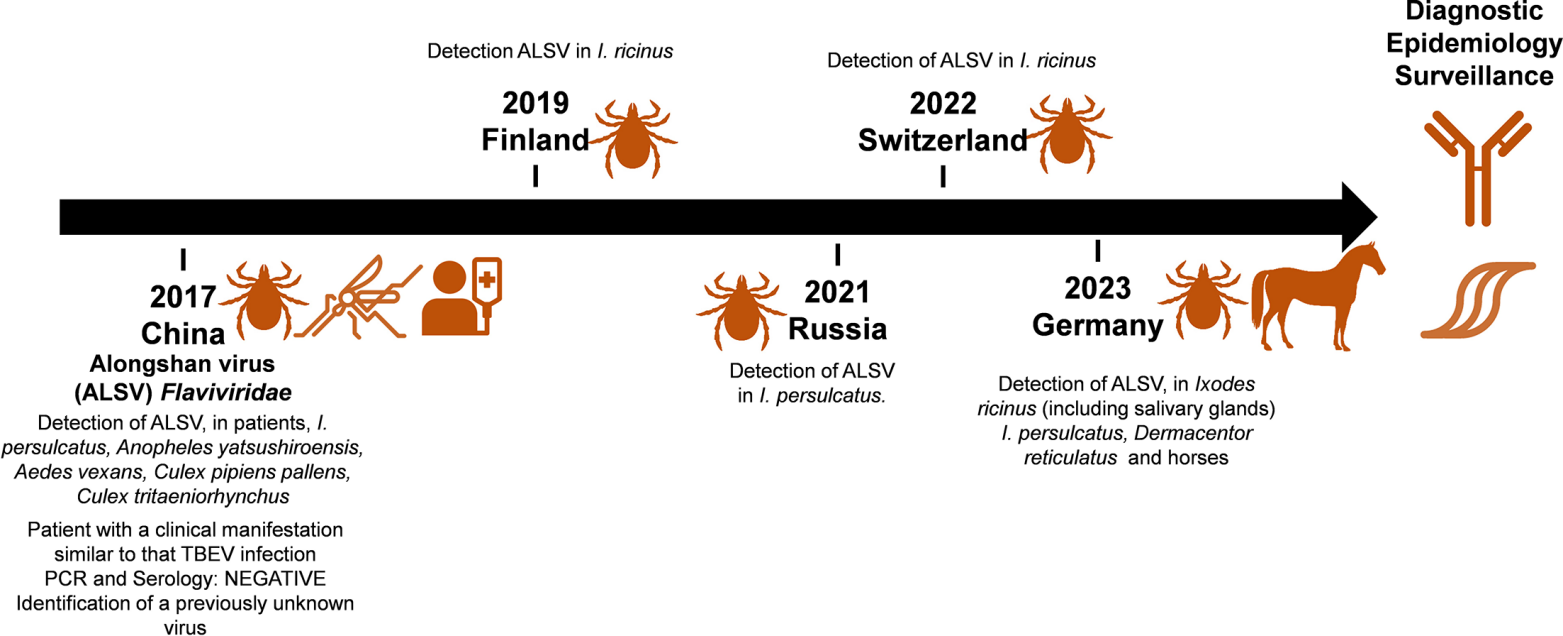
Identification and surveillance of ALSV, a member of the JMV group within the *Flaviviridae* family. ALSV has been detected in various tick species and locations from 2017 to 2023. [Wang Z-D *et al.* A new segmented virus associated with human febrile illness in China. *New England Journal of Medicine* 2019; 380 : 2116–25, Kholodilov IS, Litov AG, Klimentov AS, Belova OA, Polienko AE, Nikitin NA, *et al*. Isolation and characterisation of Alongshan virus in Russia. *Viruses* 2020; 12. Kuivanen S, *et al*. Detection of novel tick-borne pathogen, Alongshan virus, in Ixodes ricinus ticks, south-eastern Finland, 2019. *Euro Surveill* 2019; 24, Stegmueller Stefanie, Fraefel Cornel, & Kubacki Jakub. (2022). Genome sequence of Alongshan Virus from Ixodes ricinus ticks collected in Switzerland. Ebert CL, Söder L, Kubinski M, Glanz J, Gregersen E, Dümmer K, *et al*. Detection and characterization of Alongshan virus in ticks and tick saliva from Lower Saxony, Germany, with serological evidence for viral transmission to game and domestic animals. *Microorganisms* 2023; 11.]

The rapid and accurate detection of novel tick-borne viruses is critical for effective surveillance and for timely clinical management of suspected cases. In recent years, several novel viruses belonging to the Jingmenvirus (JMV) group of the family *Flaviviridae* have been described in ticks and vertebrates, including Jingmen tick virus and Alongshan virus (ALSV) [1,4]. ALSV was first described in 2017 during surveillance for tick-borne diseases in China [3]. At that time, ALSV had been considered as the possible aetiological agent of a mild tick-borne encephalitis-like illness [3]. An index patient presenting with Tick-borne encephalitis virus (TBEV)-like symptoms tested negative for TBEV RNA and was documented to be infected with ALSV. Subsequent investigations confirmed ALSV infection in several patients with fever, headache and a history of tick bites in Inner Mongolia and Heilongjiang. Furthermore, serological assays demonstrated seroconversion in patients from the acute to convalescent phase of the illness (Fig. 1). Further evidence of ALSV’s presence came from the detection of the virus in ticks. In 2020, ALSV was identified in pools of adult *Ixodes persulcatus* ticks collected in two geographically separated regions of Russia [5]. Phylogenetic analysis revealed clustering of ALSV strains with other members of the JMV group, indicating their close relationship. In southeastern Finland, ALSV was detected in *Ixodes ricinus* ticks, the same tick species transmitting TBEV [6]. However, no human cases were identified through screening of patient sera for ALSV RNA and antibodies, neither in Russia nor in Finland (Fig. 1). ALSV was then recently detected in Switzerland [7]. Next-generation sequencing of ticks collected in the Canton of Grisons unveiled the complete ALSV genome. Importantly, the detection frequency of ALSV was found to be higher than that of TBEV, raising concerns about their potential impact on human health (Fig. 1) [7,8].

Given the possible similarity of symptoms between ALSV and TBEV, we aimed to develop reliable diagnostic tools for the accurate and timely detection of ALSV infections. Molecular diagnostic platforms in reference laboratories and university teaching hospitals are equipped with various technologies for PCR-based infectious disease diagnostics. These platforms often offer a high number of PCR assays for the detection of viruses, bacteria, fungi and parasites from clinical samples. Many of these assays utilize real-time PCR on standardized automated open platforms, which are based on TaqMan technology and allow for easy in-house development [9]. The versatility and scalability of these platforms are crucial, as they can be quickly adapted to respond to emerging pathogens such as Severe acute respiratory syndrome coronavirus 2 (SARS-CoV-2) [10,11]. Overall, the comprehensive range of PCR assays available on these molecular diagnostic platforms enables accurate and efficient diagnosis of various infectious diseases, contributing to timely diagnostics, effective patient management and implementation of necessary public health measures.

ALSV is a segmented, positive-sense single-stranded RNA virus. Its four genome segments include two that encode proteins homologous to flavivirus non-structural proteins NS3 (helicase–protease) and NS5 (RNA-dependent RNA polymerase), while the remaining segments encode structural proteins unique to Jingmenviruses. This genomic architecture and the observed sequence diversity motivate the use of dual-target molecular assays directed at conserved portions of NS3 and NS5. ALSV exhibits some genetic diversity, as evidenced by variations in strains detected in different locations, including China, Russia, Finland and Switzerland. This diversity necessitates the design of diagnostic assays capable of targeting conserved regions across its genome, for instance, the NS3 and NS5 genes as targets for real-time quantitative PCR (qPCR) assays. These genes were chosen for their critical roles in the viral life cycle and their relatively conserved nature, making them ideal targets for sensitive and specific diagnostic assays.

The present study focuses on the implementation of a dual real-time PCR assay to precisely detect ALSV, on an automated molecular diagnostic platform to offer rapid, sensitive and specific detection of this virus both from various human clinical specimens, including cerebrospinal fluid (CSF) and blood, as well as insect vector specimens. This study represents a customization of existing PCR assays for ALSV detection, building upon previously described assays [3,7]. The new assays incorporate updates to account for the genetic diversity of ALSV, including recent sequences from Swiss ticks, ensuring comprehensive detection of known variants. These adaptations were validated *de novo* to ensure high specificity and sensitivity for both human clinical samples and tick specimens, while maintaining alignment with the technical requirements of our automated molecular diagnostic platform. By integrating these assays into a scalable platform, a syndromic testing approach is facilitated, enabling the simultaneous detection of ALSV and other encephalitis-associated pathogens, such as HSV1 and 2, TBEV and WNV. This implementation improves diagnostic accuracy and efficiency for both clinical and epidemiological applications, while acknowledging and advancing the original assay design. This study describes the comprehensive process of ALSV real-time qPCR assay optimization, implementation and validation and its potential in clinical specimens from patients with suspected TBEV disease. This assay holds promise for (i) its usage in epidemiological surveillance of ALSV, both in *Ixodes* spp. ticks and in other possible reservoirs, and for (ii) improving clinical management of ALSV-associated diseases if the pathogenic role of ALSV turns out to be proven.

## Methods

### Study design, sample acquisition and positive controls

We validated two ALSV-specific real-time qPCR assays (targets: NS3 and NS5) and evaluated analytical performance using positive control plasmids consisting of plasmids containing the target sequence (Table 1), which were obtained from RD-Biotech (Besançon, France), a specificity panel of clinical and external-quality-assessment specimens, banked clinical CSF and ALSV-positive tick extracts. For specificity, we tested 41 specimens positive for pathogens relevant to CSF and/or oropharyngeal samples (including 19 viruses, 7 flaviviruses, 15 bacteria, 1 fungus and 1 parasite). In addition, we tested 30 banked CSF samples received in January–February 2023 from patients with suspected meningitis/encephalitis in the Lausanne region, Switzerland, when local tick exposure is minimal. ALSV-positive tick extracts originated from the Swiss study of *I. ricinus*.

**Table 1.** NS3 and NS5 primers and probes Please note that all NS3 primers and probes need to be used all in the same cupule to perform the validated real-time qPCR; NS5 primers and probes should be used in a distinct PCR reaction.

Target gene	Primer/probe	Type	Sequence 5′−3′	Fluorochrome, Quencher	Reference	Tm	Expected Length
NS3 gene	NS3_PRIMER_F_VD1_LNA	F	TAATGGTGGCT{A}AACACATCAAACA		This study, adapted from Wang *et al*. 2019 [3]	58.1	
	NS3_PRIMER_R_CH1_LNA	R	GCATCC{A}GGTCATAATTG*GC*		This study, adapted from Wang *et al*. 2019 [3]	57.3	
	NS3_PRIMER_R_CH2_LNA	R	GCATCC{A}GGTCATAGTTA*GC*		This study, adapted from Wang *et al*. 2019 [3]	57.3	
	NS3_PRIMER_R_CH3_LNA	R	GCATCC{A}GGTCATAGTTG*GC*		This study, adapted from Wang *et al*. 2019 [3]	57.3	
	NS3_P(VIC)	P	CCTTACACCACCATCGTGCTAAGC	FAM, BHQ	Wang *et al*. 2019 [3]	64.4	
	NS3_P(VIC)_CH1	P	CCCTACACCACCATCGTGCTGAGC	FAM, BHQ	This study, adapted from Wang *et al*. 2019 [3]	67.8	
	NS3_P(VIC)_CH2	P	CCCTACACCACCATCGTGCTAAGC	FAM, BHQ	This study, adapted from Wang *et al*. 2019 [3]	67.8	
	NS3_P(VIC)_CH3_LNA	P	CCCTACACCACC{A}TCGTACTAAGC	FAM, BHQ	This study, adapted from Wang *et al*. 2019 [3]	64.4	
	Insert used for the NS3 plasmid*	UAAUGGUGGCUAAACACAUCAAACAGA -CUACCCCCUACACCACCAUCGUGCUAAGCA -GGAAAACGUAUGAGAGAAACAUCAAGCACCA -CUUCAAGCAGUACCCGAGGGGUAUGUGCGUU -GUGACAACUUCCAUCAGCGAGUGUGGCGCCAAU -UAUGACCUGGAUGC					166
NS5 gene	NS5_ALSV_S1_6_F_ZH	F	ACATGGGGCTGGTCAGAAAG		Stegmüller *et al*. 2023 [7]	59.3	
	NS5_ALSV_S1_6_R_ZH	R	TCACCGGC{A}TGTAATGGACC		This study, based on Stegmüller *et al*. 2023 [7]	57.3	
	NS5_ALSV_S1_6_R_ZH_VD1	R	TCACCGGC{A}TGTAATGGACT		This study, based on Stegmüller *et al*. 2023 [7]	57.3	
	NS5_ALSV_S1_6_P_CH	P	TGCTCACACCAGTATTGGCCGGT	FAM, BHQ	This study, based on Stegmüller *et al*. 2023 [7]	66.5	
	Insert used for the NS5 plasmid*	ACAUGGGGCUGGUCAGAAAGGA -CAUGGGAUUGGAAC AGCUCAGAGAGAUAAUCGCGGAUGUC -AGGGAGAUCAGCUUCUGCUCACACCAGU -AUUGGCCGGUAAGAUAUGGAGAUGAAGUC -CAUUACAUGCCGGUGA					135

F, forward primer; R, reverse primer; P, TaqMan probe.

Customization to match the genetic diversity of ALSV: blue and underlined.

Customization to match the technical characteristics of our in-house TaqMan automated molecular diagnostic platform: {} Locked nucleic acids and italic-green nucleotides.

*Underlined nucleotides indicate modifications made to recognize the plasmid in case of suspected contamination.

### Nucleic acid extraction

Total nucleic acids were extracted on a MagNA Pure 96 instrument (Roche) using the kit MagNA Pure 96 DNA and Viral NA Small Volume.

### Master mix, primers, probes, plasmids, positive and negative controls

The ALSV-specific real-time qPCR assays were adapted from existing PCRs (i) to meet the requirements of our automated molecular diagnostic platform, allowing for the detection of viral, bacterial, parasite and fungal nucleic acids on the same 384-well plate using TaqMan probe technology (Applied Biosystems), and (ii) to meet the criteria for diagnostic use on human clinical samples [12]. These assays targeted two different genes: NS3 and NS5. The PCR targeting the NS3 gene was adapted from the work of Wang *et al.* [3]. The PCR targeting the NS5 gene was adapted from Stegmüller *et al.* [7] (Table 1). The real-time qPCR assays for ALSV detection underwent technical adaptations to enhance their performance and ensure broad applicability. Notably, primer and probe sequences were refined to account for the genetic diversity of ALSV, as demonstrated by the blue-highlighted modifications in Table 1. A total of 40 NS3 sequences and 36 NS5 sequences downloaded from the National Center for Biotechnology Information (NCBI) were used for alignment and primer design with the Geneious software. *In silico* specificity testing was performed using the blast tool (NCBI). These adaptations were made to align with newly identified genetic variations in ALSV strains, ensuring the assays can accurately detect all currently known variants. Additionally, to address the challenge of low melting temperatures (Tm) in the original primers (below 60 °C), locked nucleic acid (LNA) modifications were incorporated, effectively raising the Tm to ~60 °C for primers. This adjustment is crucial for improving binding specificity and assay robustness, minimizing non-specific amplification and ensuring consistent diagnostic reliability. In addition, LNA increased the Tm, which allows the assays to be run in combination with other assays on the same 384-well plate and enhances PCR stability at low DNA copy numbers per reaction. These combined technical adaptations enhance the specificity, sensitivity and robustness of the assays, making them well-suited for clinical and epidemiological applications (Table 1). We tested several concentrations of primers and probes and observed the obtained amplification curves at various positive control concentrations. This optimization for TaqMan real-time qPCR assays was performed to balance sensitivity, specificity and curve geometry on the Thermo Fisher QuantStudio^™^ Pro. Primers and probe were titrated to maximize early exponential growth and plateau amplitude while minimizing baseline noise, using serial 10-fold dilutions to target an efficiency of ~90–110% (slope ≈ −3.1 to −3.6). Under optimal conditions, amplification curves showed clean sigmoidal traces with parallel log-linear phases across dilutions, stable ROX-normalized baselines, tightly clustered and early Cq values, endpoint ΔRn around 1, and flat no nucleic acid template. After optimization, the concentration of primers and probes was 0.9 and 0.3 µM for the NS3 real-time qPCR and 0.6 and 0.2 µM for the NS5 real-time qPCR. The qPCR master mix, TaqPath 1-Step real-time qPCR Master Mix, was purchased from Thermo Fisher and used in a final volume of 20 µl, including 5 µl of RNA extract. Simplex assays were run in parallel and in three replicates for each assay to increase the analytical sensitivity.

### Real-time qPCR conditions, instruments and programme

Our automated molecular diagnostic platform utilized in this study includes the following components: the MagNA Pure 96 instrument (Roche) for nucleic acid extraction, the STARletR instrument (Hamilton, Cinnaminson, USA) for liquid handling distribution, two Hamilton instruments for assembling the 384-well PCR plates and two QuantStudio 7 instruments (Applied Biosystems, Waltham, USA) [9]. The PCR programme used on our platform involved the following temperature specifications for annealing and synthesis: During the hold, step 1 was set at 25 °C for 2 min, step 2 at 50 °C for 7 min and step 3 at 95 °C for 10 min. The real-time qPCR stage consisted of 45 cycles, with step 1 at 95 °C for 1 s and step 2 at 60 °C for 20 s. The reactions were performed on a 384-well plate using a QuantStudio 7 ‘fast’ thermocycler.

### Analytical sensitivity, specificity and reproducibility of the real-time qPCR

The analytical performance of each real-time qPCR assay was evaluated individually using the corresponding positive control plasmids, consisting of plasmids containing the target sequence (Table 1), which were obtained from RD-Biotech (Besançon, France). These synthetic plasmids were used to determine the analytical limit of detection and the reproducibility of the real-time qPCR [12,14]. The analytical sensitivity was evaluated using serial dilutions (1,000, 100, 10, 1 and 0.1 copies per reaction) of the positive control plasmids. Detection frequencies at each concentration were used to estimate performance; the limit of detection (LoD) was defined *a priori* as the lowest concentration with ≥95% positive replicates. The intra-run variability was evaluated by plotting the cycle threshold (Ct) values of the 5 replicates from the same amplification run using plasmid dilutions of 100 and 10 copies per reaction, as previously described. Similarly, the inter-run reproducibility was assessed in five independent runs using dilutions of the plasmids corresponding to 100 and 10 copies per reaction. The synthetic plasmids were also used in each run of analysis to draw the standard curve for quantification and to assess assay efficiency. With the QuantStudio Pro, each amplification run was accompanied by a standard curve generated from reactions containing 10, 100 and 1,000 plasmid copies. The ideal slope is −3.32, corresponding to 100% efficiency, while values between −3.0 and −4.0 are considered acceptable. The slope is mainly influenced by the lowest positive control (10 copies), whose variability has a direct impact on the calculated efficiency. For the specificity, both real-time qPCRs were tested using DNA from closely related organisms and from unrelated organisms that can colonize or infect the same body sites as ALSV. The specificity of the assays was tested using clinical specimens and external quality control samples (EQC). TBEV strain Neudoerfl, WNV, YFV 17D, DENV type-2 and ZIKV were kindly provided by Olivier Engler, Spiez Laboratory, Switzerland. TBEV was grown on A549 cells, WNV, YFV and ZIKV on Vero cells and DENV on BHK-21 cells. When cells displayed extensive cytopathic effect, the supernatant was collected and clarified by centrifugation for 10 min at 1,500 r.p.m. The supernatant was then inactivated and subjected to real-time qPCR as described above. The sensitivity of the two PCRs was subsequently assessed using tick samples previously confirmed to be ALSV-positive.

### Data, data analysis and statistical analysis

The data for this study were collected as part of a quality enhancement project conducted at our institution. In accordance with national regulations, the project’s performance and publication of results can be carried out without seeking permission from the relevant research ethics committee.

Ct values and detection frequencies were analysed in Excel and GraphPad Prism 8.3 (GraphPad Software, San Diego, CA, USA, https://www.graphpad.com/). Cts were analysed using Bland–Altman analysis.

## Results

### Assay design and optimization for an open real-time qPCR platform

To ensure compatibility with our platform’s harmonized cycling conditions, we optimized primer/probe concentrations and incorporated LNA modifications where required to adjust Tm while maintaining specificity. Primer and probe sequences were also modified to encompass the genetic variability of ALSV documented in the meantime. To achieve this, we relied on all available ALSV sequences from the NCBI, including the latest sequences from Swiss tick samples (Table 1).

### Analytical sensitivity and reproducibility

**Fig. 2.**
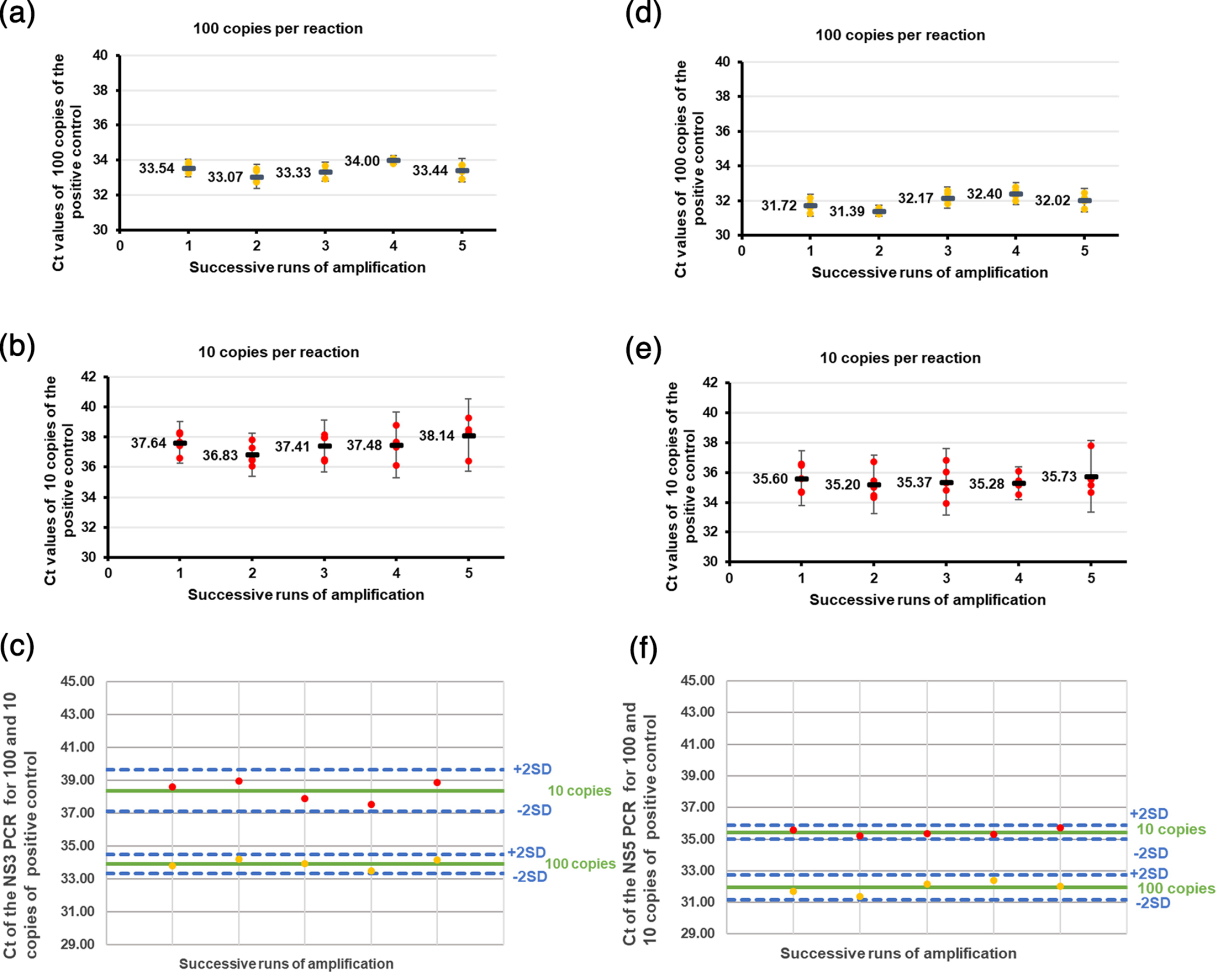
Reproducibility of the NS3 and NS5 real-time qPCRs. Intra-run variability of the NS3 real-time qPCR (**a, b**) and NS5 real-time qPCR (**d,e**). The average and dispersion of the five replicates are shown for each amplification run, with error bars indicating two standard deviations (2SD) around the mean. Inter-run variability using 10 and 100 plasmid copies per reaction obtained during five successive runs for the NS3 real-time qPCR (**c**) and NS5 real-time qPCR (**f**). The solid black lines represent the mean Ct values, and the dashed lines (on both sides of the means) indicate 2SD. Each concentration was tested in 25 replicates (5 runs×5 amplifications), and every run was accompanied by a standard curve generated from 10, 100 and 1,000 plasmid copies (ideal slope −3.32; acceptable range −3.0 to −4.0). Amplification plots and standard curves are shown in Fig. S1.

Analytical sensitivity and reproducibility were estimated using the synthetic plasmids. During each run amplification, we also used dilutions of the plasmid at 1,000, 100 and 10 copies per reaction to draw the standard curve for quantification. Detection frequencies for NS3 real-time qPCR were 100%, 100%, 92%, 72%, 20%, 28 and 0% at 1,000, 100, 10, 5, 2, 1 and 0.1 copies per reaction, respectively. For NS5, the detection frequencies were 100%, 100%, 92%, 88%, 40%, 20% and 0% at the same concentrations (Table 2). Based on the ≥95% definition, LoD was 100 copies per reaction for both real-time qPCR. However, as the real-time qPCRs are performed in triplicate on our platform, the LoD can be estimated at five copies per reaction for the NS3 real-time qPCR and two copies per reaction for the NS5 real-time qPCR. For both real-time qPCRs, the intra-run variability analysis showed that the dispersion of each replicate remained within two standard deviations of the average (Fig. 2). The inter-run variability analysis demonstrated that the dispersion of the average Ct value for each run did not exceed two standard deviations of the overall average across all runs (Figs 2 and S1, available in the online Supplementary Material).

**Table 2.** Limit of detection of the NS3 and NS5 real-time qPCR

Copies per reaction (plasmid)	Frequencies of amplification using the NS3 real-time qPCR in %	Frequencies of amplification using the NS5 real-time qPCR
1,000	100 (25/25)	100 (25/25)
100	100 (25/25)	100 (25/25)
50	100 (25/25)	100 (25/25)
20	100 (25/25)	100 (25/25)
10	92 (23/25)	92 (23/25)
5	72 (18/25)	88 (22/25)
2	20 (5/25)	40 (10/25)
1	28 (7/25)	36 (9/25)
0.5	8 (2/25)	32 (8/25)
0.2	4 (1/25)	8 (2/25)
0.1	0 (0/25)	0 (0/25)

Using *a priori* definition of limit of detection (LoD) as ≥95% positive replicates, LoD was 100 copies per reaction for both real-time qPCRs. However, as the real-time qPCRs are performed in triplicate on our platform, the LoD can be estimated at five copies per reaction for the NS3 real-time qPCR and two copies per reaction for the NS5 real-time qPCR.

### Specificity of the NS3 and NS5 real-time qPCRs

Both real-time qPCRs were tested using DNA from closely related organisms and from unrelated organisms that can be present in CSF and/or in oropharyngeal samples, where ALSV could be searched for. Firstly, specificity was tested on 36 positive clinical and external quality control samples for various pathogens, including 19 viruses, of which 7 were flaviviruses. Additionally, 15 bacteria, 1 fungus and 1 parasite were also tested. No amplification was obtained for the samples positive for these 41 pathogens (Table 3). The specificity test was then extended to 30 clinical samples corresponding to negative CSF samples received in our laboratory between 23 January 2023 and 23 February 2023, from patients with suspected meningitis, encephalitis or meningoencephalitis. This period was chosen as tick-borne encephalitis is not prevalent in western Switzerland during that time of year. All ALSV NS3 and NS5 real-time qPCRs returned negative results, as expected (Table 3). In total, 71 samples were tested for the specificity of both real-time qPCRs, and these negative results strongly support the high specificity of the developed real-time qPCRs.

**Table 3.** Specificity and sensitivity of the NS3 and NS5 real-time qPCR on CSF, clinical specimen and external quality control positive for various pathogens

Specificity of the ALSV real-time qPCR			
Pathogens found in the specimen		NS3 real-time qPCR	NS5 real-time qPCR
**Total**	**41**	**Negative**	**Negative**
**Virus**	19	Negative	Negative
Flavivirus*	12	Negative	Negative
VZV	3	Negative	Negative
Enterovirus	2	Negative	Negative
HSV1	2	Negative	Negative
HSV2	2	Negative	Negative
CMV	1	Negative	Negative
HHV6	1	Negative	Negative
JC Virus	1	Negative	Negative
**Bacteria**	15	Negative	Negative
*Staphylococcus aureus*	2	Negative	Negative
*Streptococcus agalactiae*	2	Negative	Negative
*Haemophilus influenzae*	2	Negative	Negative
*Streptococcus pneumoniae*	2	Negative	Negative
*Treponema pallidum*	1	Negative	Negative
*Listeria monocytogenes*	1	Negative	Negative
*Escherichia coli*	1	Negative	Negative
*Mycobacterium tuberculosis*	1	Negative	Negative
*Tropheryma whipplei*	1	Negative	Negative
*Mycoplasma pneumoniae*	1	Negative	Negative
*Neisseria meningitidis*	1	Negative	Negative
**Fungi**	1	Negative	Negative
*Cryptococcus neoformans*	1	Negative	Negative
**Parasite**	1	Negative	Negative
*Toxoplasma gondii*	1	Negative	Negative
**Negative CSF from January to February 2023**			
CSF1 to CSF 30	30	Negative	Negative
**Sensitivity of the ALSV real-time qPCRs**			
**Tick positive for ALSV**		**NS3 real-time qPCR**	**NS5 real-time qPCR**
**Tick 1 dilution 1**	1	Positive Ct 30.856	Positive Ct 28.805
**Tick 1 dilution 2**	1/10	Positive Ct 34.320	Positive Ct 32.231
**Tick 1 dilution 3**	1/100	Positive Ct 37.215	Positive Ct 35.821

*Flavivirus (*n*=12):

West Nile virus (EQC, *n*=3; infected cell culture lysate, *n*=1).

Usutu virus (EQC, *n*=2).

Tick-borne encephalitis virus (infected cell culture lysate, *n*=1).

Yellow fever virus (infected cell culture lysate, *n*=1).

Dengue virus (infected cell culture lysate, *n*=1).

Zika virus (infected cell culture lysate, *n*=1).

Unknown flavivirus (EQC, *n*=2).

### Detection of ALSV in tick samples

Finally, we tested both NS3 and NS5 real-time qPCRs on ALSV-positive tick samples identified in the Swiss study [7]. After inactivating the samples in BSL3 conditions, we performed total nucleic acid extraction and conducted geometric dilutions, which were then tested using both real-time qPCR assays following the protocol developed and presented in this study. All three dilutions returned positive results for both PCR assays, with significantly lower Ct values observed for the NS5 real-time qPCR as compared to the NS3 PCR (Table 3). This suggests that these real-time qPCR assays could detect ALSV from positive tick samples, and the NS5 real-time qPCR exhibits a slightly higher sensitivity when compared to the NS3 real-time qPCR.

## Discussion

We validated two ALSV-specific TaqMan real-time qPCR assays suitable for use on open, high-throughput diagnostic platforms. These assays enable rapid and accurate identification of specific pathogens, allowing for timely response and effective control measures. Dual targeting of NS3 and NS5 mitigates the risk of false negatives arising from polymorphisms in any single region. An important design rationale was compatibility with a harmonised cycling protocol (single annealing temperature) to enable parallel testing of multiple pathogens on the same 384-well plate [9]. This molecular platform provides a flexible and customizable approach, allowing for the rapid introduction and optimization of diagnostic tests. As demonstrated in recent global health crises, such as the COVID-19 pandemic [10,11, 13] and the Monkeypox virus outbreak [15], the availability of an open molecular diagnostic platform has proven to be essential in the response to the public health threat of such emerging pathogens and to offer a diagnostic tool during the months that are needed for industry to develop new PCR assays and to obtain the necessary CE and or FDA certification [10,11, 13]. In addition to offering accelerated onboarding of assays for emerging pathogens, this approach increases throughput and reduces per-sample cost.

We aimed to validate a robust real-time qPCR diagnostic tool for the rapid detection of ALSV, a tick-borne flavivirus. We focused on two previously described ALSV-specific Taqman real-time qPCR assays, respectively, targeting the NS3 and NS5 genes [3,7]. These assays were adapted to accommodate the genetic diversity of ALSV sequences and optimized to match the technical characteristics of our in-house automated molecular diagnostic platform. This included optimizing parameters such as melting temperature by incorporating LNA modifications on certain oligonucleotides to increase their Tm and to ensure good analytical performance and increased specificity. Primers and probes were also adjusted as described above to be compatible with annealing temperature and specifications of our automated platform, which allows testing on the same 384 microplate a large number of pathogens [9]. Moreover, we checked that the new primers and probes are able to theoretically detect all ALSV variant sequences currently available on NCBI, including the latest sequences obtained from Swiss ticks. As a result, our real-time qPCRs should detect all ALSV strains, despite the observed gene target polymorphism.

Both real-time qPCR assays were evaluated for specificity using 41 DNA samples from closely related micro-organisms as well as unrelated micro-organisms known to colonize or infect the body sites where ALSV detection by real-time qPCR is expected. All 41 samples were negative. Moreover, all 30 CSF samples yielded negative results. These findings highlight the excellent specificity of the ALSV-specific real-time qPCRs.

The use of a dual targets’ detection technique is important for real-time qPCR-based approaches, especially for emerging micro-organisms or recently described viruses like ALSV. Dual targeting enhances the comprehensiveness and robustness of the assay by reducing the likelihood of false negatives and ensuring that diverse strains of the micro-organisms are detected. For the ALSV real-time qPCR, a single-target approach (e.g. targeting NS5) may fail to detect certain strains due to mutations or polymorphisms in the target sequence. By both NS5 and an NS3 region, our assay provides a broader detection capability, ensuring that even genetically diverse strains are identified; indeed, if mutations occur in one target region, the other target can still be detected, thus maintaining the reliability of the diagnostic test.

The primary benefit of our study is the validation of the real-time qPCR assay on human clinical specimens, particularly focusing on specificity. Due to the limited availability of positive specimens from patients, it is challenging to comprehensively address clinical sensitivity. However, the specificity validation supports the assay’s reliability in distinguishing ALSV from other viruses, including other flaviviruses, in human samples. In addition, clinical studies often require high-specificity assays to accurately evaluate the prevalence and impact of a pathogen.

The developed ALSV real-time qPCR assays provide a rapid, sensitive and highly specific method for the detection of ALSV infections. The integration of these assays into our automated molecular diagnostic platform enables syndromic testing, allowing simultaneous detection of ALSV and other encephalitis-associated viruses such as FSME or WNV. Indeed, the newly developed assays for ALSV detection have been successfully introduced into routine diagnostics for the investigation of encephalitis cases, providing a rapid and precise tool for detecting the virus in clinical samples. Additionally, the assays are currently being used in an ongoing retrospective study to analyse archived clinical specimens from patients with suspected encephalitis, aiming to assess the potential association of ALSV with neurological conditions. While the results of this retrospective study are not included in this manuscript, they will be presented in a forthcoming publication, further contributing to our understanding of ALSV’s clinical relevance and its potential role in human encephalitis. Moreover, the ALSV real-time qPCR assays hold great potential for high-throughput screening of ticks, facilitating effective surveillance efforts.

This study highlights the value of automated molecular diagnostic platforms in the introduction of diagnostic tests, particularly in the context of emerging micro-organisms like ALSV.

This new real-time qPCR assay will be used in the near future with a novel serological assay still under development in order to perform a broad clinical investigation to define whether or not ALSV may be associated with meningoencephalitis. The fact that ALSV is a *Flaviviridae* does not mean it shares similar pathogenicity with the TBEV and a detailed investigation is warranted since, except for a few cases described in China of possible ALSV infections, so far nobody could document a case of meningoencephalitis [5,6]. Even the Chinese cases are unlikely to have suffered from a typical meningoencephalitis, since they were promptly recovering, being all discharged from the hospital in less than 2 weeks [3].

The successful implementation of the ALSV-specific real-time qPCR assay highlights the importance of having a flexible and adaptable diagnostic platform in responding to and anticipating emerging infectious diseases. As we continue to face new challenges in the realm of infectious diseases, the utilization of open molecular diagnostic platforms plays a fundamental role and current *In Vitro* Diagnostic Regulation in Europe represents a risk by reducing at term the number of laboratories that will have the competencies and capacities to rapidly develop new real-time qPCR assays when facing future outbreaks or any possible threat due to an emerging pathogen.

## Supplementary material

10.1099/acmi.0.000917.v3 Uncited Fig. S1.

## Abbreviations

ALSV: Alongshan virus
CSF: cerebrospinal fluid
Cts: cycle thresholds
EQC: external quality control samples
JMV: Jingmenvirus
LNA: locked nucleic acid
LoD: limit of detection
NCBI: National Center for Biotechnology Information
qCPR: quantitative PCR
TBEV: Tick-borne encephalitis virus
Tm: melting temperatures
