# Supplementary figures and images for: Two TaqMan real-time quantitative PCR assays for the detection of Alongshan virus, a new member of the tick-borne Flaviviridae family
Source: Access Microbiol. 2026 Feb 9;8(2):000917.v3. doi: 10.1099/acmi.0.000917.v3 (PMC12888789; doi:10.1099/acmi.0.000917.v3)

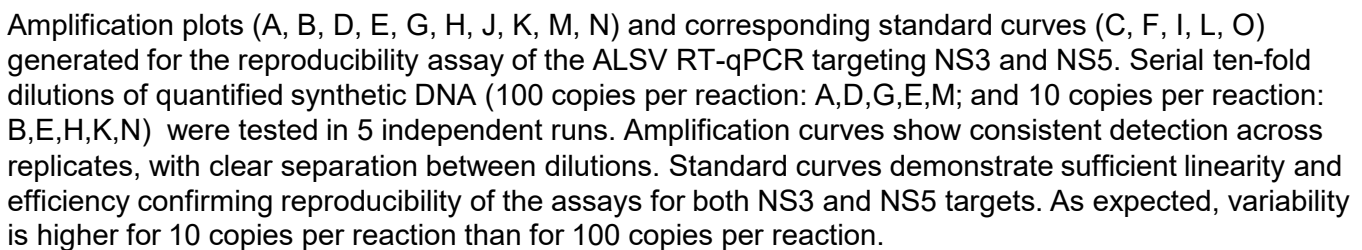

Supplement: Uncited Fig. S1. [file acmi-8-00917-s001.pdf]
